# Synergy between flow and light fields and its applications to the design of mixers in microalgal photobioreactors

**DOI:** 10.1186/s13068-019-1430-y

**Published:** 2019-04-23

**Authors:** Chao Qin, Jing Wu, Jing Wang

**Affiliations:** School of Energy and Power Engineering, Huazhong University of Science and Technology, Wuhan, 430074 China

**Keywords:** Microalgal photobioreactor, Static helical mixer, Light/dark cycle, Pumping cost, Synergy between flow and light fields

## Abstract

**Background:**

Mixers are usually inserted into microalgal photobioreactors to generate vortices that can enhance light/dark cycles of algal cells and consequently enhance biomass productivity. However, existing mixer designs are usually developed using a trial-and-error approach that is largely based on the designer’s experience. This approach is not knowledge-based, and thus little or no understanding of the underlying mechanisms of mixer design for mixing performance of photobioreactors is attained. Moreover, a large pumping cost usually accompanies mixer introduction, and this cost is not favorable for practical applications. This study aims to improve this situation.

**Results:**

In addition to the individual effects of flow and light fields, improving the synergy (coordination) between these fields may markedly enhance the L/D cycle frequency with a lower increase in pumping costs. Thus, the idea of synergy between flow and light fields is introduced to mixer design. Better synergy can be obtained if (a) the vortex core and L/D boundary are closer to each other and (b) the vortex whose core is too far from the L/D boundary is removed. The synergy idea has two types of applications. First, it can facilitate a better understanding of known numerical and experimental results about mixer addition. Second, and more importantly, the idea can help to develop new rules for mixer design. A helical mixer design is provided as a case study to demonstrate the importance and feasibility of the synergy idea. An effective method, i.e., decreasing the radial height of the helical mixer from the inner side, was found, by which the L/D cycle frequency was enhanced by 10.8% while the pumping cost was reduced by 23.8%.

**Conclusions:**

The synergy idea may be stated as follows: the enhancement of L/D cycle frequency depends not only on the flow and light fields individually but also on their synergy. This idea can be used to enhance our understanding of some known phenomena that emerge by mixer addition. The idea also provides useful rules to design and optimize a mixer for a higher L/D cycle frequency with a lower increase in pumping costs, and these rules will find widespread applications in PBR design.

**Electronic supplementary material:**

The online version of this article (10.1186/s13068-019-1430-y) contains supplementary material, which is available to authorized users.

## Background

Microalgae are used to produce pharmaceuticals, food and, looking toward the future, biofuels. Currently, large-scale cultivation of microalgae occurs mainly in open ponds. However, open ponds consume much water, and algae in these ponds are easily contaminated. Researchers invented enclosed photobioreactors (PBRs) to address these problems. Enclosed PBRs can be generally divided into tubular PBRs, bubble columns, flat-plate PBRs and so on. Among these PBRs, tubular PBRs have been recognized for their potential to cultivate algae on a large scale because they can cultivate and harvest cells continuously [1, 2].

Designers are always seeking ways to increase algal biomass productivity in PBRs, and the way of introducing mixers to PBRs (including open ponds) has been well developed over the past several decades, such as the trapezoidal chamber mixer in a flat-plate PBR [3], the up–down chute baffles in an open pond [4], the Kenics mixer [5], the plate mixer with a V-groove at the top and a hole at the center [6–8], the inclined porous mixer [9] and the helical mixer [10] in tubular PBRs. Apart from mixers, novel structures (e.g., the discrete double-inclined ribs in a tubular PBR [11] and a wavy bottom in a pond-like PBR [12]) have also been introduced to PBRs to increase biomass productivity. These novel structures actually serve as mixers for enhancing PBR mixing performance.

The reason why the introduction of mixers can increase biomass productivity can be understood as follows: On the one hand, the mixing effect caused by the mixer accelerates the gas exchange between the cells and the medium. On the other hand, the vortices generated by mixers improve the convection between the light and dark zones in light-limited cultures and, therefore, enhance the frequency of light/dark (L/D) cycles of algal cells. Evidence for the relationship between the L/D cycle frequency and biomass productivity has been reported many times. Many studies [3, 13–19] have indicated that an increase in L/D cycle frequency may lead to an increase in microalgal biomass productivity because of the flashing light effect (FLE) [16, 20, 21], and Huang et al. [3] further concluded by correlation analysis of experimental and simulation results that the L/D cycle frequency is a parameter that directly relates to the biomass output.

The development of mixers has mostly been based on trial-and-error approaches. Namely, after proposing a novel mixer, the performance of a PBR with this novel mixer is compared with that of a PBR with an existing mixer (or that of a PBR without mixers) in terms of one or more measurable quantities (e.g., biomass productivity). If the former performs better than the latter, the novel mixer is retained. Otherwise, it is discarded to start a new design. For example, in 2013, Zhang et al. [10] proposed a helical mixer, and the biomass productivity of a PBR with this type of mixer increased by 37% compared to that of a smooth tubular PBR without mixers. Therefore, they introduced this helical mixer into tubular PBRs. Recently, aware of the fact that the use of a helical mixer largely increased the pumping cost of a PBR (~ 2105.9%) [1, 22], Gómez-Pérez et al. designed a new mixer shape (i.e., a twisted tube [22]) and introduced it into a tubular PBR.

Likewise, the design of geometrical parameters of existing mixers has also been mainly based on such trial-and-error approaches. That is, after assigning a new value to the geometrical parameter, if the new value makes a PBR with the mixer achieve better performance, the old value is replaced by the new value. Otherwise, the old value is retained. An example showing this process can be found in Ref. [23].

However, such trial-and-error approaches depend on the designer’s experience to a large extent. In some cases, designers have to process each shape or parameter value of a mixer individually within a certain range to identify the best value to be used. This approach is rather time-consuming and thus impractical for a cost-effective design.

Several research teams have tried to obtain a unified principle that may generally guide mixer design. von Boxtel’s team [1, 22] has designed mixers by following the idea of achieving lower pressure loss with acceptable mixing performance. The parameters they used to evaluate mixing performance were the autocorrelation function [1], swirl number and frequency calculated by discrete Fourier transform taking simulated cell trajectories as a signal. However, there is little evidence of a direct relationship between the autocorrelation function and biomass productivity. Moreover, the swirl number or frequency calculated by discrete Fourier transform does not include the influence of light, one of the most important nutrients for microalgae. Li’s team [3, 9, 23] has designed mixers by following the idea of achieving a higher average velocity along the light direction. However, the detailed ways to achieve such a velocity have not been discussed. The mixer design process in their work was still on a trial-and-error basis.

From the above description, there are two disadvantages of existing methods for PBR mixer design. First, these methods lack a way to understand the inherent universal mechanism of various mixer designs for mixing performance enhancement. Second, a large additional flow resistance, and hence the pumping cost, usually accompanies the introduction of a mixer and is not favorable for practical applications.

In this paper, a synergy (coordination) idea is introduced into PBR mixer design. This idea is proposed based on one of the growth characteristics of microalgae—the FLE. As mentioned above, an increase in the L/D cycle frequency may contribute to an increase in microalgal biomass productivity. The parameters of flow and light are crucial in a reactor design. However, in some cases, the obtained value of the L/D cycle frequency may not be as high as expected, although the flow and light conditions appear to be individually satisfactory. The reason is analyzed in detail in “Synergy mechanism between flow and light fields” section, and this reason indicates that the L/D cycle frequency in PBRs with a mixer depends not only on the flow and light fields but also on their synergy. The applications of the synergy idea to PBR mixer design are then presented. Consequently, we not only can have a better understanding of the universal mechanism of various mixer designs but also can further develop novel mixers or structures that can enhance the L/D cycle frequency with a small or reasonable increase in the pumping cost.

## Methods

### Evaluation parameters of mixers

Before a discussion of the synergy idea, we list and briefly discuss the basic evaluation parameters of mixers used in this study.

#### Absolute vorticity flux

In this study, the vortex intensity in PBRs is analyzed to facilitate the discussion in “L/D cycle frequency” section on how changes in the geometrical parameters of a helical mixer affect the L/D cycles. The vortex intensity can be characterized by the absolute vorticity flux [24], defined as

1 $$J_{\text{ABS}} = \frac{{\mathop {\iint }\nolimits_{s}^{{}} \left| {\omega_{z} } \right|{\text{d}}S}}{S}$$

where *J*_ABS_ is the absolute vorticity flux, *ω*_z_ is the axial component of vorticity and *S* is the cross-sectional area of PBRs.

#### The statistics of the L/D cycle

Luo and Al-Dahhan [25] calculated L/D cycles by the binary L/D pattern [5]. This pattern splits the light field into two zones—light zone and dark zone—and ignores the differences within each zone. The light zone is where the local light intensity is higher than the critical light intensity, while the dark zone is where the local light intensity is lower than the critical light intensity [25]. The critical light intensity is the saturated light intensity [25–27]. For *Chlorella pyrenoidosa*, this value is approximately 580 foot candles [28], which is approximately 96.84 μmol m^−2^ s^−1^ [3]. Thus, 96.84 μmol m^−2^ s^−1^ is assumed to be the critical light intensity in this study.

A complete duration of the L/D cycle is [3, 25, 26]

2 $$t_{\text{c}} = t_{\text{d}} + t_{\text{l}} ,$$

where *t*_d_ and *t*_l_ are the durations in which a particle stays in the dark and light zones, respectively. The L/D cycle frequency is

3 $$f = 1/t_{\text{c}}$$

The mean duration of L/D cycles of each particle is [3]

4 $$t_{{{\text{c}},{\text{av}}}}^{\text{ID}} = \frac{{\mathop \sum \nolimits_{1}^{n} t_{\text{c}} }}{n},$$

where *n* is the number of L/D cycles an individual particle has experienced and ID is the serial number of the particle [29]. There are particles that have not experienced L/D cycles during their lifetimes. To include these particles in the calculation of the average frequency, their *t*_c_ is assumed to be their lifetimes [30].

It is necessary to calculate a large number of particles to eliminate the randomness of the results since the particle trajectory model is based on the Gaussian probability distribution [3, 29]. In this work, 1000 particles are selected as recommended in Ref. [3], and verification is discussed in Additional file 1. The average L/D cycle of the particle group [3] (the average of the mean duration of L/D cycles of each individual particle) is

5 $$t_{{{\text{c}},{\text{av}}}} = \frac{{\mathop \sum \nolimits_{{{\text{ID}} = 1}}^{{{\text{ID}} = N}} t_{{{\text{c}},{\text{av}}}}^{\text{ID}} }}{N},$$

where *N* is the total number of these particles [3]. By substituting *t*_c,av_ into Eq. 3, we can obtain the average L/D cycle frequency as

6 $$f_{\text{av}} = 1/t_{{{\text{c}},{\text{av}}}}$$

#### Pumping cost

The pumping cost per unit time of the PBR is calculated by [1]

7 $$E = \phi \Delta P,$$

where $\phi$ is the volumetric flow rate of the algal suspension and $\Delta P$ is the total pressure drop. These values are determined by

8 $$\phi = U_{\text{av}} S$$

9 $$\Delta P = P_{\text{up}} - P_{\text{down}} ,$$

where *U*_av_ is the average inlet velocity and *P*_up_ and *P*_down_ are the average total pressures at the surface where particles are released and the surface at *z *= 1.5 m, respectively. *P*_up_ and *P*_down_ are given by

10a $$P_{\text{up}} = \frac{{\mathop {\iint }\nolimits_{s}^{{}} \left( {P_{{{\text{s}},{\text{local}}}} + P_{{{\text{d}},{\text{local}}}} } \right){\text{d}}S}}{S}$$

10b $$P_{\text{down}} = \frac{{\mathop {\iint }\nolimits_{s}^{{}} \left( {P_{{{\text{s}},{\text{local}}}} + P_{{{\text{d}},{\text{local}}}} } \right){\text{d}}S}}{S},$$

where *P*_s, local_ and *P*_d, local_ are the local static pressure and dynamic pressure, respectively [1, 29].

#### Efficiency of L/D cycle enhancement

The efficiency of L/D cycle enhancement is a newly defined quantity inspired by the efficiency concept, whose essence is the ratio of produced valuable resources to consumed ones, reflecting how effectively the input is converted to the output [29]. This concept provides a possible way to evaluate the enhancement of L/D cycles and the increase in pumping costs simultaneously caused by a mixer. The efficiency is a ratio of the dimensionless increment of the L/D cycle frequency to the dimensionless increment of the pumping cost per unit time, namely,

11 $$\eta = \frac{{\Delta f_{\text{av}} /f_{{{\text{av}},0}} }}{{\Delta E/E_{0} }},$$

where *f*_av,0_ and *E*_0_ are the L/D cycle frequency and pumping cost per unit time of a PBR without mixers (smooth PBR), respectively, and $\Delta f_{\text{av}} = f_{\text{av}} - f_{{{\text{av}},0}}$ and $\Delta E = E - E_{0}$ are the increment of the L/D cycle frequency and the pumping cost per unit time, respectively, of a PBR with mixers relative to those of the smooth PBR. Here, the smooth tubular PBR serves as the benchmark for the evaluation of both the enhancement of the L/D cycle frequency and the increase in the pumping costs. In this work, the flow rate of the algal suspension in the PBR is constant, and hence, we have $\Delta E/E_{0} = \left( {\Delta P - \Delta P_{0} } \right)/\Delta P_{0}$, namely, the ratio of the pumping costs is equal to the ratio of the pressure drop [29].

### Synergy mechanism between flow and light fields

**Fig. 1:**
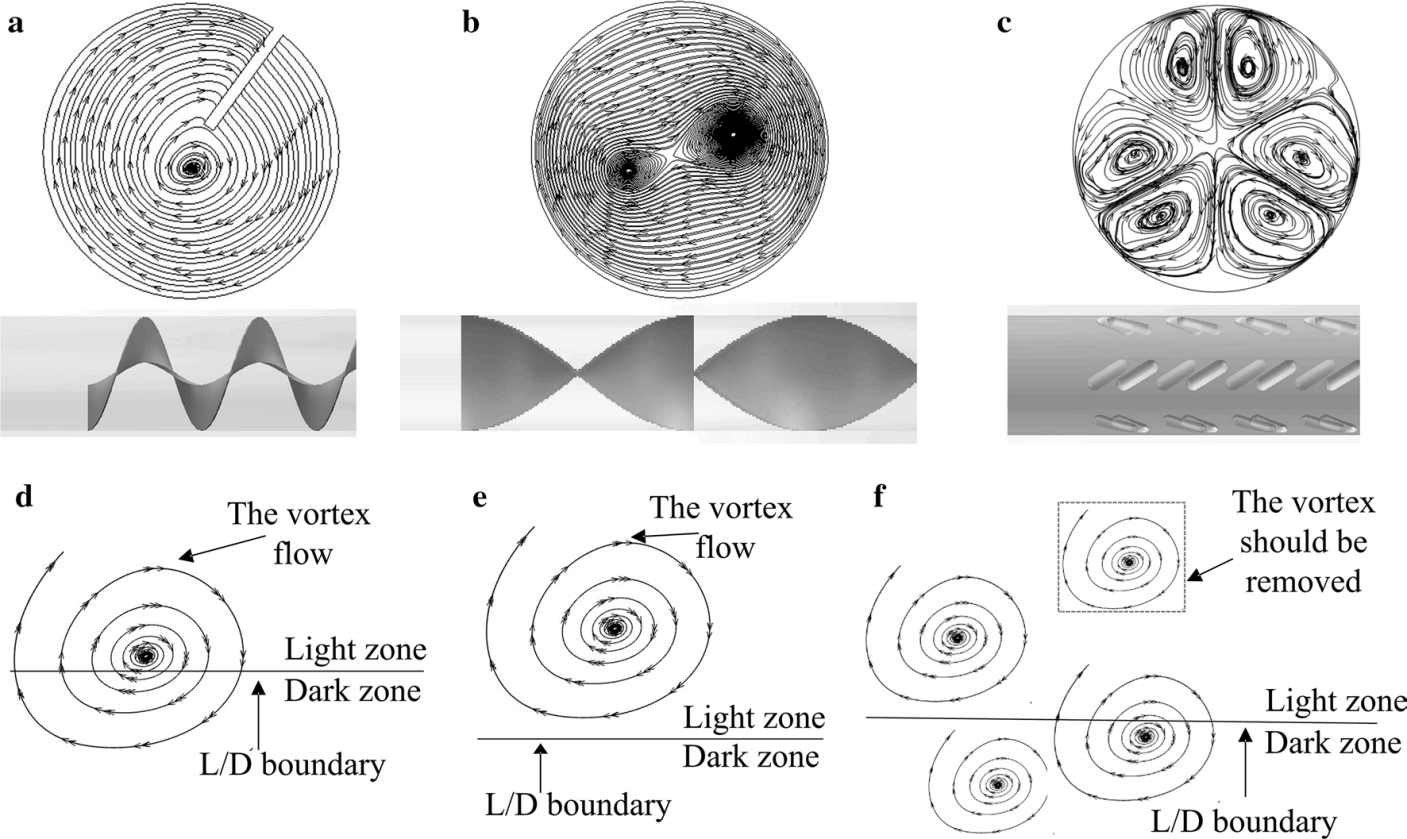
**a**–**c** Typical vortices induced by a helical mixer, Kenics mixer and discrete double-inclined ribs, respectively. **d**–**f** The scheme of a vortex whose core is located at the L/D boundary, a vortex whose core is located far from the L/D boundary and a vortex that should be removed, respectively

As mentioned in the background section, mixers generate vortices (three examples are shown in Fig. 1a–c). The vortices guide the fluid rotating in the PBR rather than that flowing straight forward. As the L/D cycles are accomplished by back and forth movements of cells across the light/dark zones, these vortices can enhance L/D cycles, as illustrated in Fig. 1d.

Now, let us imagine a case as shown in Fig. 1e. The vortex is the same as the one in Fig. 1d, but its core is located far from the L/D boundary (where the local light intensity is equal to the critical light intensity, as mentioned in “Synergy mechanism between flow and light fields” section). In this case, although the movements of the cells near the vortex core are enhanced to a large extent by the vortex, the potential of the vortex to increase the L/D cycle frequency has not been fully exploited because these cells are far from the L/D boundary. As a consequence, there might be only a small fraction of the back and forth movements induced by the vortex that could move across the L/D boundary to enhance the L/D cycles. In addition, due to a lack of a driving effect by the vortex, it is difficult for the algal cells near the L/D boundary (i.e., far away from the vortex core) to generate very strong movements back and forth between the light and dark zones (i.e., to generate many L/D cycles). Thus, the L/D cycles for the case shown in Fig. 1e would be less than those for the case shown in Fig. 1d.

In some cases (e.g., Fig. 1c), there may be several vortices in a cross section of the PBR. In such cases, removing the vortex whose core is too far from the L/D boundary (Fig. 1f) could save pumping costs because the viscous and turbulent dissipation of energy will be lessened as the number of vortices decreases. Moreover, doing so may have little negative effect or even a positive effect on the enhancement of L/D cycles since the vortex core is too far from the L/D boundary.

The analysis above indicates that, in addition to the flow and light conditions, there is another important factor that influences the value of the L/D cycle frequency—the relative locations of the vortex cores to that of the L/D boundary. In some cases (e.g., the case shown in Fig. 1e), a vortex core is located far from the L/D boundary, and consequently, the obtained value of the L/D cycle frequency may not be as high as expected although the flow and light conditions appear to be individually satisfactory. The L/D cycle frequency could be enhanced with as little pumping cost as possible if (a) the vortex core and L/D boundary are closer to each other and (b) the vortex whose core is too far from the L/D boundary is removed. However, the flow field determines the vortex characteristics (such as number of vortices, intensity and position), while the light field determines the light characteristics (such as light intensity and the L/D boundary). To obtain results (a) and (b), the positions of the vortex cores and the L/D boundary line have to be coordinated. That is, a concerted action between the flow and light fields is needed. As “synergy is two or more things functioning together to produce a result not independently obtainable” [31], we name this concerted action “synergy”.

The synergy idea may be stated as follows: the enhancement of L/D cycle frequency depends not only on the flow field and the light field but also on their synergy. A well-designed mixer for a PBR may indeed play the role of a “helper” for such synergy. As a mixer is inserted into a PBR, the original flow field of the PBR will change. If the mixer is well-designed, the new flow field makes the positions of the vortex cores coordinate well with that of the L/D boundary to obtain results (a) and (b) as much as possible. Consequently, insertion of a well-designed mixer increases the L/D cycle frequency of a PBR without greatly increasing the pumping costs. A detailed case study of a concrete mixer design is presented in “Applying the synergy idea to the design of a helical mixer: a case study” section. Additionally, a change in the light field (e.g., by changing the incident light) may also contribute greatly to the coordination of the positions of the vortex cores and the L/D boundary, and this effect is also discussed in “Applying the synergy idea to the design of a helical mixer: a case study” section.

## Results and discussion

### Applications of the present synergy idea

The synergy idea has two types of applications. First, the idea can facilitate a better understanding of known numerical and experimental results about mixer addition. Second, and more importantly, the idea can help to develop new rules for mixer design. In the following, two examples are provided to show the first type of application (“Further understanding of results about mixer addition” section), and then, focus is paid to the second type of application, including a brief description (“Developing new rules for mixer design” section) and a detailed case study (“Applying the synergy idea to the design of a helical mixer: a case study” section).

#### Further understanding of results about mixer addition

**Fig. 2:**
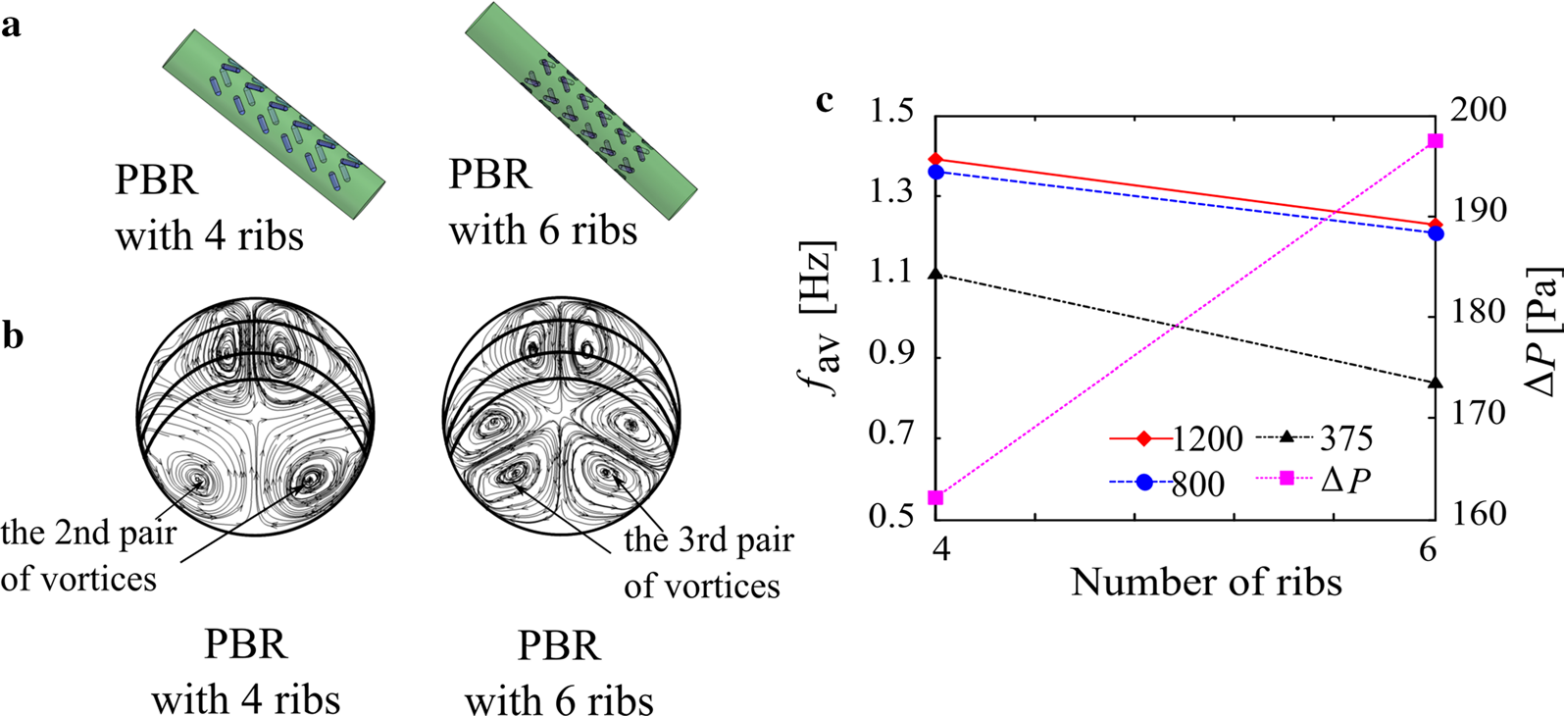
**a** Schemes of PBRs with 4 and 6 discrete inclined ribs, **b** the vortices in cross sections of these two PBRs, and **c** the L/D cycle frequency and pressure drop of these two PBRs [11]; legends (except ∆*P*) in the figure are incident light intensities; for example, 1200 represents an incident light intensity of 1200 μmol m^−2^ s^−1^

Qin et al. [11] studied the performance of tubular PBRs with 4 and 6 discrete double-inclined ribs (Fig. 2a) by numerical method. The pressure drop of the PBR with 4 ribs was lower than that of the PBR with 6 ribs (Fig. 2c). The physical mechanism lies in the fact that as the number of ribs decreases from 6 to 4, the pairs of vortices formed in the cross section of the tube correspondingly decrease (Fig. 2b), leading to lower turbulent intensity and resulting in lower pumping costs. Moreover, as shown in Fig. 2c, the L/D cycle frequency of the PBR with 4 ribs is higher than that of the PBR with 6 ribs. This result may be because in the latter case, the movements of the cells near the cores of the third pair of vortices have little benefit to the enhancement of the L/D cycle since these cores are far away from the L/D boundary. However, as this pair of vortices is removed, the cells in this domain can generate many more L/D cycles due to the driving effect of the second pair of vortices. As a result, a higher L/D frequency is obtained.

Huang et al. [3, 23] conducted a numerical and experimental study of a trapezoidal chamber mixer in a flat-plate PBR. They found that as the chamber was moved closer to the illuminated surfaces, the L/D cycle frequency and biomass productivity increased, while the pumping costs decreased [23]. This phenomenon can be well explained based on the synergy between flow and light fields. In this case, the L/D boundary is close to the illuminated surface because the incident light intensity, 540 μmol m^−2^ s^−1^, is not very high. As the chamber moves closer to the illuminated surfaces, the vortices induced by the mixers move closer to the illuminated surfaces (as shown in Fig. 2 of Ref. [23]), and as a result, the L/D frequency increases.

#### Developing new rules for mixer design

Mixer design can be generally categorized as novel mixer/structure design and geometrical parameter design for an existing mixer/structure. In novel mixer/structure design, we first obtain the position of the L/D boundary in a PBR by using the light transfer model. Then, we design the mixer/structure by the following rules: (a) the cores of each vortex induced by the mixer/novel structure should be near the L/D boundary as much as possible, and (b) the mixer/novel structure should not generate unnecessary vortices that are too far from the L/D boundary.

In geometrical parameter design for an existing mixer/structure, we first obtain the position of the L/D boundary by using the light transfer model and the vortex distribution in a PBR with the mixer/structure through the velocity and pressure fields. Then, we adjust the geometrical parameter by the following rules: (a) if most of the vortex cores are far from the L/D boundary, there is much room for improvement open to the existing mixer/structure; otherwise, if most of the vortex cores are near the L/D boundary, the room for improvement is limited; (b) determine the key geometrical parameter among several ones that governs the position of the vortex cores in relation to the L/D boundary based on the distribution and development of vortices in the PBR; and (c) find the proper value of the geometrical parameter such that the cores of each vortex induced by the mixer/structure with this parameter value are closer to the L/D boundary and there are less vortices too far from the boundary. Such a nearly optimal value is searched for through several possible values.

It is worth noting that the synergy idea gives us the direction (or more detailed rules) to improve the L/D cycle frequency with less additional pumping costs. However, the specific configuration of the novel mixer/structure or the specific geometrical parameter value of an existing mixer/structure that can make better synergy for a given condition cannot be obtained from the idea itself. Finding such a mixer/structure/value is an individual research task. This distinction, of course, by no means implies that the idea is not useful; rather, the idea is very useful, as shown above, and is demonstrated by a design example presented below.

### Applying the synergy idea to the design of a helical mixer: a case study

Zhang et al. reported that the addition of a helical mixer increased the biomass productivity of *Chlorella* sp. by 37% [10]. In their experiment, the length of the PBR was 200 m, the largest scale among all the tubular PBRs with mixers to the authors’ knowledge, implying that a helical mixer is appropriate in large-scale microalgae cultivation. Moreover, this type of mixer is easy to manufacture and install. Thus, in this section, we use the geometrical parameter design of a helical mixer as an example to illustrate the importance and feasibility of the synergy idea.

#### The tubular PBR and inserted helical mixer

**Fig. 3:**
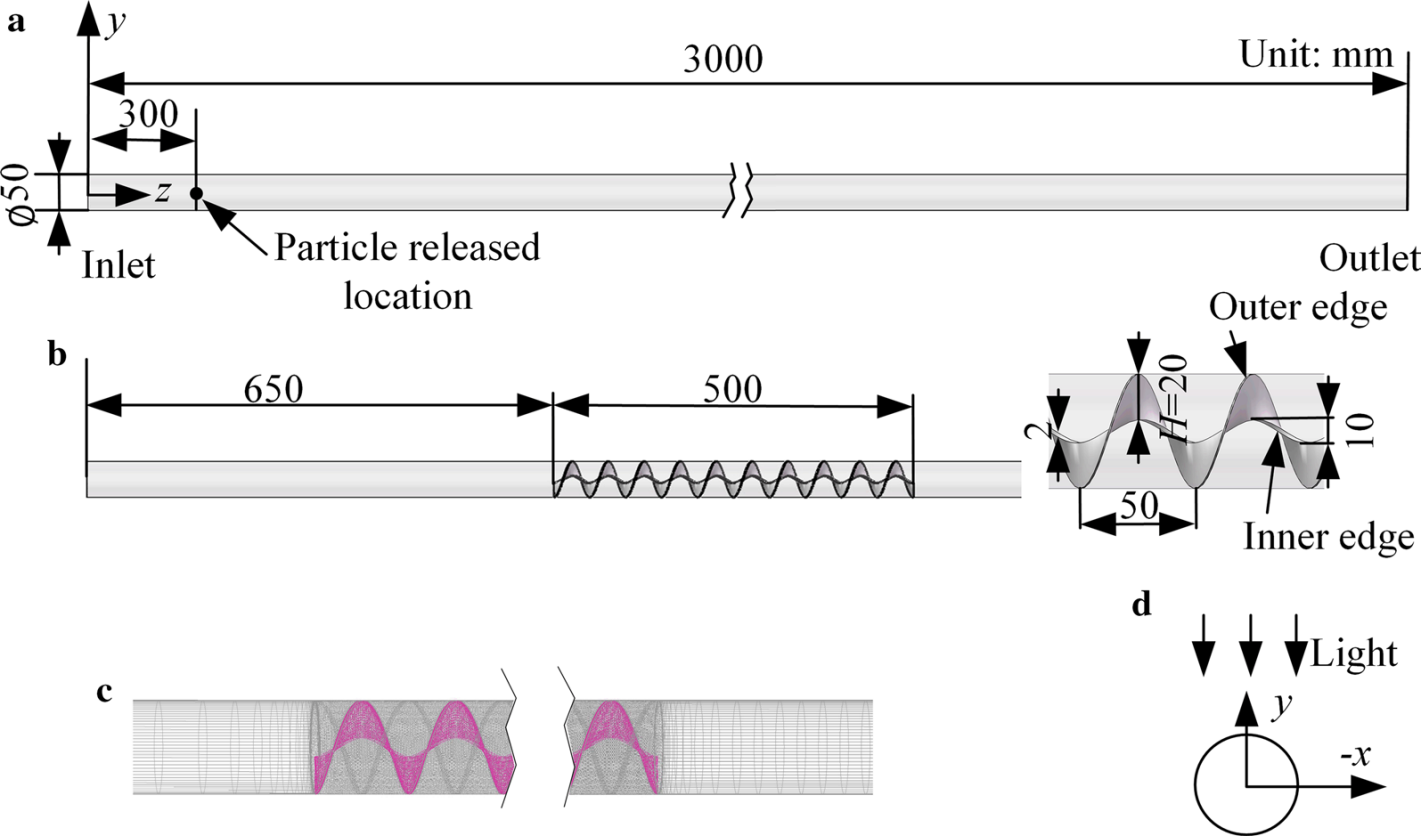
**a** Smooth PBR, named H0, **b** PBR with a helical mixer with *H *= 20 mm, named H20, **c** the mesh of H20, **d** direction of the incident light

Figure 3a shows the geometry of the tubular PBR. The inner diameter of the pipe, *D*, is 0.05 m, as recommended for large-scale outdoor applications [32, 33]. The simulated tubular length is 3 m. Figure 3b shows the geometry of the helical mixer. Its radial height, *H*, is 20 mm. There is no clearance between the outer edge of the mixer and the pipe. The tubular PBR with this mixer is named H20. Its thickness is 2 mm, and the pitch is 50 mm. The first 0.65 m of the pipe is considered a blank zone where the flow develops before entering the mixer zone, and the last 1.5 m is not included in the analyses of this work but only contributes to iteration convergence. A structured mesh (Fig. 3c) is generated by ICEM CFD (ANSYS Inc., USA). The regions of the boundary layers and two ends of the mixer are locally refined based on the evaluation of *y*^+^ (nondimensional distance from the cell centers of the first layer grid to the wall; a detailed description of *y*^+^ can be found in Ref. [34]) to capture steep gradients near the walls and to satisfy the wall treatment model used in this work. The simulation methods for turbulence and particle trajectories are the same as those in Ref. [29].

Light is assumed to be along the −*y* direction (Fig. 3d) and transfers only forward and backward, so the light distribution is the same at different cross sections along the *z* direction [9]. The materials of the pipe and mixers are assumed to be transparent, and the impacts of these materials on the light transfer are negligible [3, 5, 9]. The light transfer model is the Cornet model, and the parameters are the same as those in Ref. [29], except that the incident light intensity range in this work is much wider than that in Ref. [29] and the biomass density is 1.3 g L^−1^ in this work.

#### Methods to achieve better synergy in PBRs with helical mixers

**Fig. 4:**
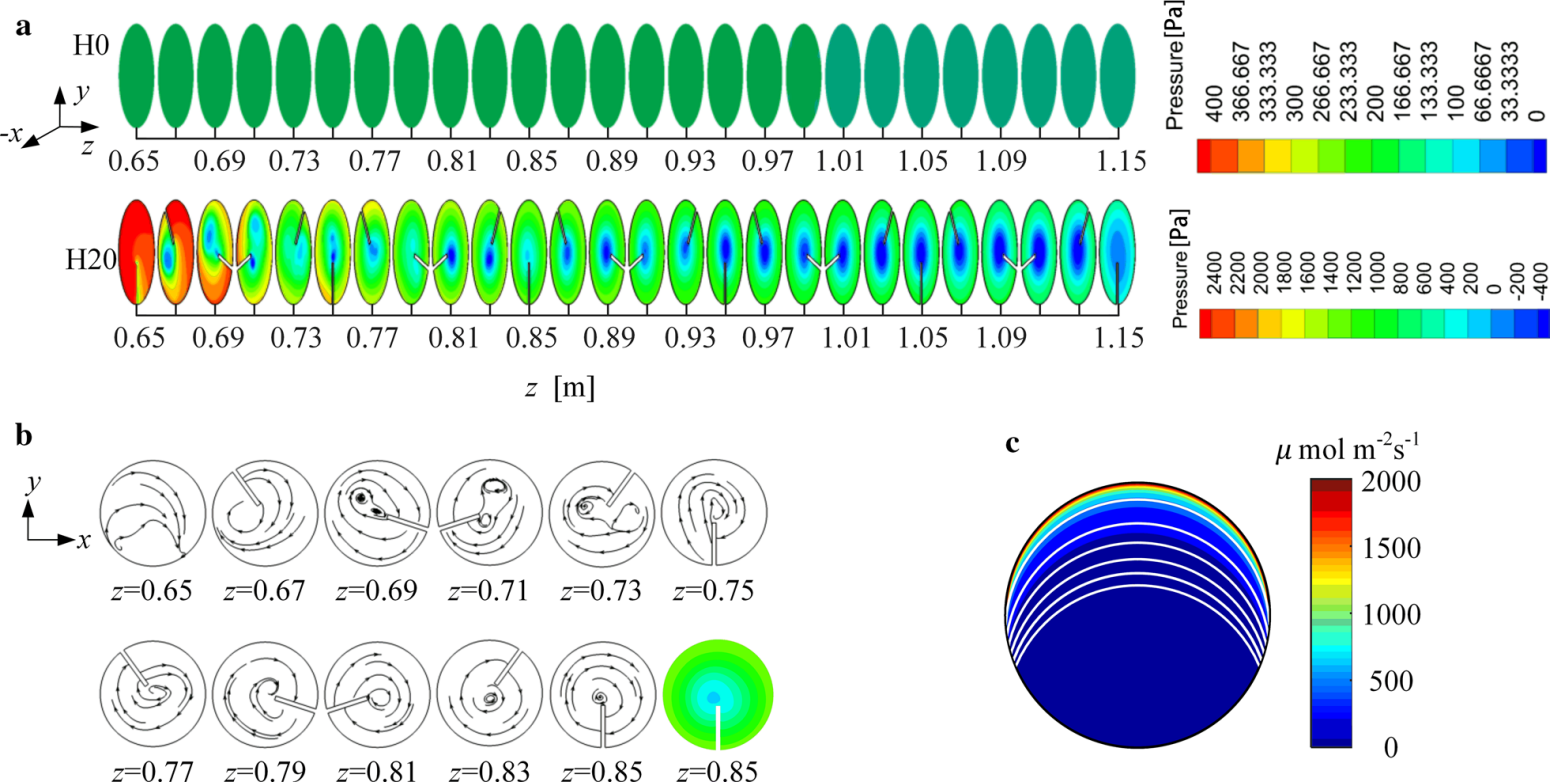
**a** The pressures at 26 axial locations (0.65 m ≤ *z* ≤ 1.15 m) in H0 and H20 along the flow direction (*z *-axis), **b** streamlines at 11 axial locations (0.65 m ≤ *z *≤ 0.85 m) in H20; the last subfigure shows the pressure at the axial location of *z* = 0.85 m for a clear view, **c** light field calculated from the Cornet model at an incident light intensity, *I*_0_, of 2400 μmol m^−2^ s^−1^, where the white lines represent the L/D boundaries for *I*_0_ = 375, 800, 1200, 1600, 2000 and 2400 μmol m^−2^ s^−1^ from top to bottom

The positions of the vortices can be shown by the pressure field since the vortex cores are usually located in regions where the pressure is lower than that in the surrounding fluid [35, 36]. We examined the pressure fields in a smooth PBR (Fig. 3a, named H0) and a PBR with a helical mixer (Fig. 3b, named H20). The results are shown in Fig. 4a. For a convenient comparison, only the pressure field in the domain where the helical mixer is positioned (from *z* = 0.65 m to *z* = 1.15 m) is shown here. The streamlines in a cross section of H20 are also plotted to visually display the vortex flow, as shown in Fig. 4b. Note that the streamlines in the domain of *z* > 0.85 m are not included in this figure because the vortex patterns in each cross section of this domain are quite similar to each other, as shown in Fig. 4a.

Figure 4c shows the results of the light profile in the PBR at *I*_0_ = 2400 μmol m^−2^ s^−1^ as an example. The white lines represent the L/D boundaries (i.e., the boundaries of L/D zones) at different incident light intensities (*I*_0_ = 375, 800, 1200, 1600, 2000, and 2400 μmol m^−2^ s^−1^ from top to bottom).

In the smooth PBR without a mixer, the pressure field in each cross section of H0 is uniform, namely there is no vortex in the smooth PBR (Fig. 4a). In this case, the L/D cycles are dominated by the random turbulent vibrations of the algal cells [25], and thus, the L/D cycle frequency of the smooth PBR is generally not very high. In contrast, the mixer in H20 can induce vortices (Fig. 4a, b) that enhance the movements of the algal cells back and forth across the L/D boundaries in light-limited cultures. As a result, a much higher L/D cycle frequency is obtained. As shown in Fig. 4a, b, two vortices are generated near *z* = 0.69 m, and then they quickly merge into a single vortex near *z* = 0.75 m. This single vortex becomes stable near *z* = 0.81 m.

However, there is still room for enhancement of the L/D cycle frequency of H20 in view of the synergy between the flow and light fields. As shown in Fig. 4a, b, once a single vortex is formed in H20, the vortex core remains in the center region of the pipe. This position is far from the L/D boundary, especially in the cases of relatively low incident light intensities (Fig. 4c). Thus, making the vortex and L/D boundary closer to each other is required to increase the L/D cycle frequency.

There are two ways to make the vortex and L/D boundary closer to each other. One is adjusting the position of the L/D boundary. This position is determined by the light condition of the PBR. As shown in Fig. 4c, as the incident light intensifies, the L/D boundary moves closer to the center region of the pipe, the position where the vortex core is located. Thus, increasing the incident light intensity may be a viable solution to achieve better synergy between the flow and light fields. In addition, the light field can also be affected by the cell density. As the cell density increases, the light attenuation becomes faster and the L/D boundary moves closer to the illuminated surface. This trend is similar to the effect of decreasing the incident light intensity on the L/D boundary position.

The other way is relocating the vortex. The position of the vortex core in a PBR is dominated by the structure of the mixer. The structure of a helical mixer is determined by its radial height, screw pitch and thickness. The thickness of the mixer depends on the material used in the manufacture. Moreover, the screw pitch largely affects the vortex flow along the axial direction of the pipe and has a relatively small impact on the positions of the vortices along the radial direction, according to the results in a study of a Kenics mixer [37]. Thus, the screw pitch is not the primary design parameter in this study, considering that the purpose of our design based on the synergy idea is to relocate the vortices along the radial direction to approach the L/D boundary. In contrast, the radial height of the helical mixer may be the key geometrical parameter that governs the positions of the vortex cores along the radial direction since the vortex seems to be shed from the inner edge of the mixer, as shown in Fig. 4b. There is only one single vortex induced by the helical mixer. This finding means that removing the vortex to save energy is not an available option here. However, the purpose of energy savings can be achieved by reducing the radial height of the mixer [10].

**Fig. 5:**
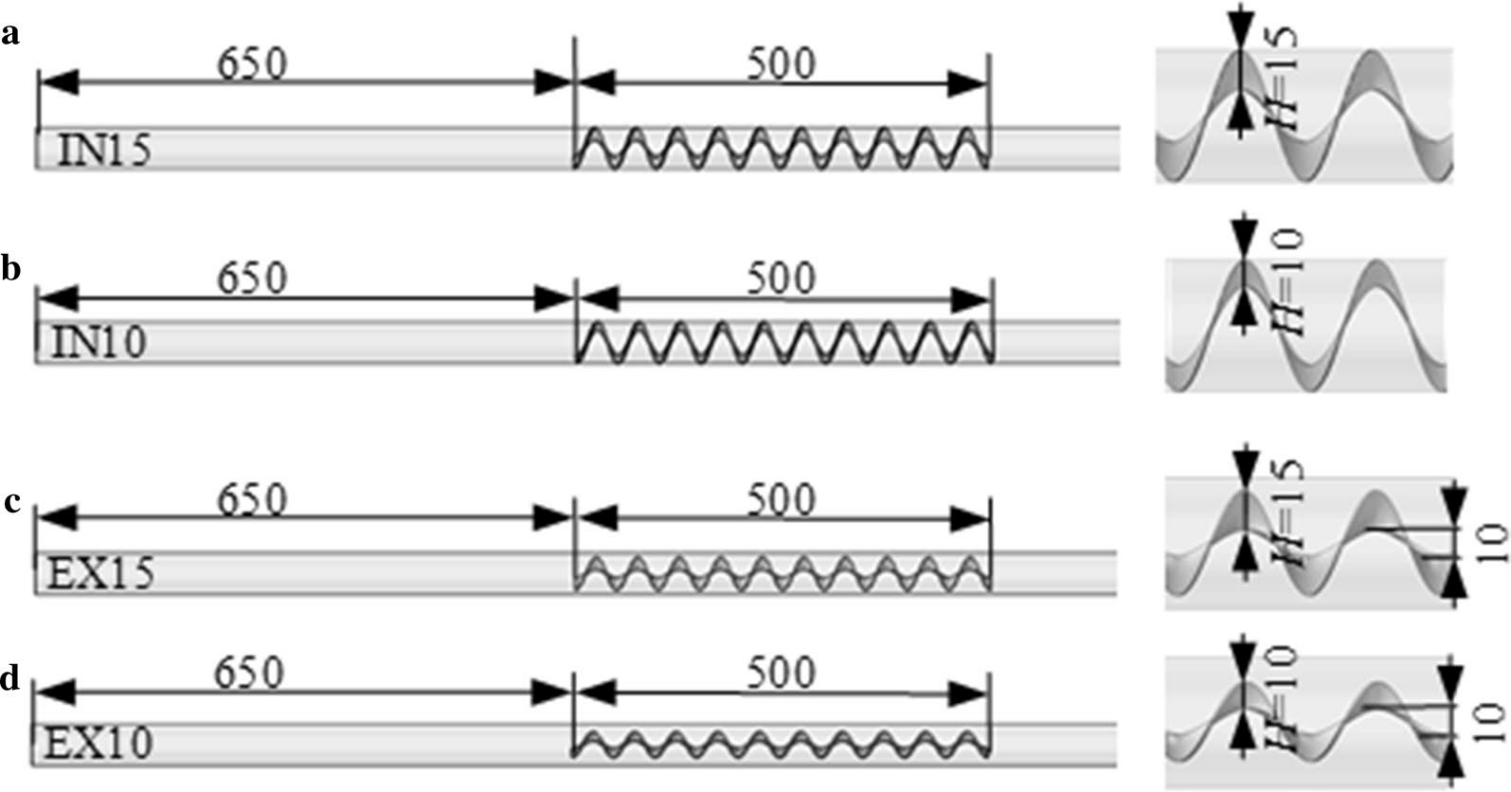
PBR with a helical mixer with **a** *H* = 15 mm, named IN15 (*H* is decreased by 5 mm from the inner side), **b** *H* = 10 mm, named IN10 (*H* is decreased by 10 mm from the inner side), **c** *H* = 15 mm, named EX15 (*H* is decreased by 5 mm from the outer side), **d** *H* = 10 mm, named EX10 (*H* is decreased by 10 mm from the outer side)

Four structures of the helical mixer with different radial heights are considered, as shown in Fig. 5a–d. For the two structures shown in Fig. 5a, b, *H* is decreased by 5 mm and 10 mm, respectively, from the inner side, while for the two structures shown in Fig. 5c, d, *H* is decreased by 5 mm and 10 mm, respectively, from the outer side. The corresponding tubular PBRs with these four mixers are named IN15, IN10, EX15 and EX10, respectively. The thickness and screw pitch of the helical mixer remain unchanged, and their values are the same as those in Fig. 3b.

#### Results of the synergy idea applied to the helical mixer design

##### Flow structure

**Fig. 6:**
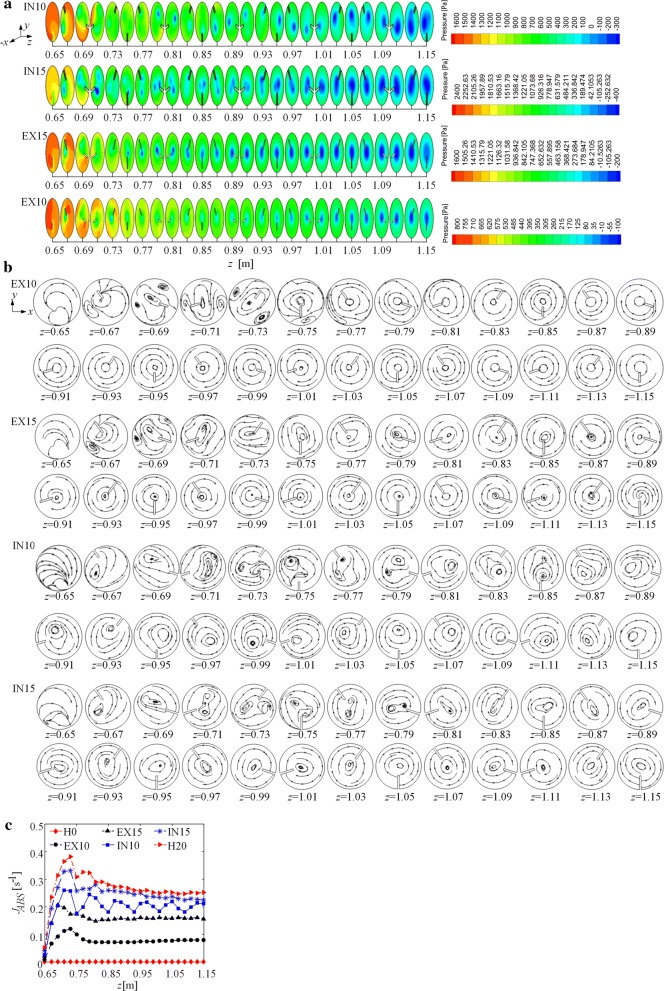
**a** The pressures at 26 axial locations (0.65 m ≤ *z* ≤ 1.15 m) in EX10, EX15, IN10 and IN15 along the flow direction (*z*-axis); the legend levels in these cases are scaled to show the vortex cores in each case, **b** streamlines at 26 axial locations (0.65 m ≤ *z* ≤ 0.85 m) in EX10, EX15, IN10 and IN15, **c** variation in absolute vorticity flux, *J*_ABS_, in six PBRs along the flow direction

The pressure fields and the streamlines in the cross sections of the four PBRs are shown in Fig. 6a, b. As shown in Fig. 6b, the vortices in EX10 and EX15 merge into a single vortex near *z* = 0.79 m and 0.75 m, respectively. Then, the vortex becomes stable, and its core is located in the center region of the pipe. In contrast, in IN10 and IN15, the vortices merge into a single vortex near *z* = 0.87 m and *z* = 0.83 m, respectively, indicating that the multivortex structure remains over a longer distance in IN10 and IN15 than in EX10 and EX15. Furthermore, at *z* = 1.15 m (the end of the mixer), the vortex in IN10 and IN15 is still oval, which means that the merging of vortices is still on-going there [38].

As shown in Fig. 6a, b, the vortex cores in H20, EX15 and EX10 are located in quite similar positions, that is, the center region of the pipe, and remain in this region along the entire domain with a helical mixer, although the radial heights of the mixers in EX15 and EX10 have been decreased by 5 mm and 10 mm from the outer side, respectively. This result indicates that the change in the radial height of the helical mixer from the outer side generally has a low impact on the relocation of the vortices. Compared with EX15 and EX10, the vortex cores in IN15 and IN10 are located closer to the wall of the pipe. This difference means that reducing the radial height of the helical mixer from the inner side is an effective way to relocate the vortex cores. Moreover, the vortex cores in IN10 are located farther from the core region of the pipe than those in IN15 (Fig. 4b), implying that the more the radial height of the mixer is decreased from the inner side, the farther the vortex cores are located from the core region of the pipe.

The single vortex structure in H20 and the result that the vortex core in H20 is located in the pipe core region are consistent with the flow patterns reported in Refs. [10, 22, 39]. Moreover, the positions of the single vortex core in EX15 and EX10 are also consistent with the flow pattern reported in Ref. [39]. However, the structure of a pair of vortices has not yet been reported. The reason may be that a mixer (or a whole PBR) design based on the flow structure has not drawn much attention so far.

The absolute vorticity flux, *J*_ABS_, of a cross section of the pipe is calculated according to Eq. 1, and the variation of this flux along the flow direction is plotted in Fig. 6c. This figure shows that *J*_ABS_ decreases as the radial height of the helical mixer decreases, namely, the vortex intensity decreases as the radial height decreases (from both the inner and outer sides). This finding means that a decrease in the radial height leads to a weaker vortex flow. This trend with respect to the vortex intensity is applied to the analysis of the L/D cycle frequency.

##### L/D cycle frequency

**Fig. 7:**
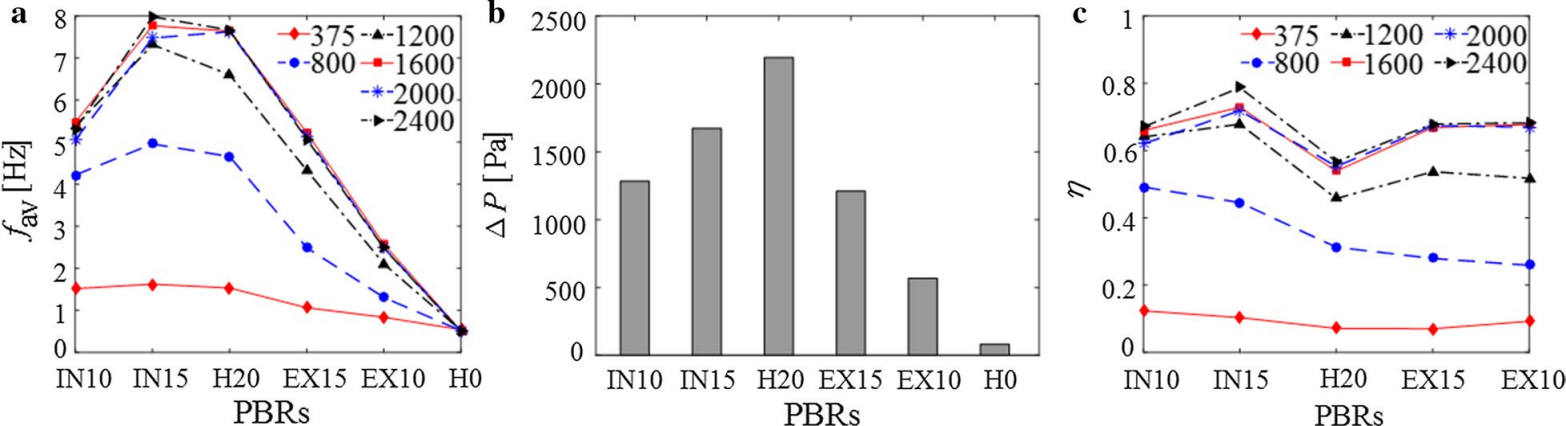
**a** Average L/D cycle frequency *f*_av_ of PBRs with radial height reduced from the inner side and outer side, **b** pressure drop, ∆*P*, of the six PBRs, **c** efficiency of L/D cycle enhancement, *η*, for the six PBRs. The values in the legends represent incident light intensity (for example, 375 is the case of *I*_0_ = 375 μmol m^−2^ s^−1^)

The average L/D cycle frequency, *f*_av_, of the six PBRs (H0, H20, IN10, IN15, EX10, and EX15) at different incident light intensities is calculated by Eq. 6, and the results are shown in Fig. 7a.

As shown in Fig. 7a, for each PBR, *f*_av_ clearly increases as the incident light intensity increases from 375 μmol m^−2^ s^−1^ to 1200 μmol m^−2^ s^−1^, verifying that increasing the incident light intensity is a viable solution to achieve better synergy between the flow and light fields and, consequently, to enhance the L/D cycle frequency, as noted in “Synergy mechanism between flow and light fields” section. However, as the incident light intensity increases further (from 1200 to 2400 μmol m^−2^ s^−1^), the increase in *f*_av_ is no longer obvious. The reason can be understood by the relative positions of the L/D boundary and the vortices. As shown in Fig. 4c, the L/D boundaries at these three intensities are very close to each other and are all located near the core region of the pipe, where the vortices are located. Because of this effect, the synergy between the flow and light fields is not improved significantly by increasing the light intensity from a high to a higher value.

The results for H20 and IN15 shown in Fig. 7a demonstrate that as the radial height of the helical mixer decreases from the inner side by 5 mm, *f*_av_ increases under a wide range of incident light intensities (by 6.0%, 6.4% and 10.8% under *I*_0_ = 375 μmol m^−2^ s^−1^, 800 μmol m^−2^ s^−1^ and 1200 μmol m^−2^ s^−1^, respectively). These increases can be explained by two aspects. First, as the radial height decreases from the inner side, the vortices move closer to the L/D boundaries (Fig. 7b). These vortices could play an important role in moving particles across light and dark zones and, consequently, increase the L/D cycle frequency. Second, the multivortex structure remains over a longer distance in IN15 than in H20 (in IN15 and H20, the vortices merge into a single vortex near *z* = 0.83 m and *z* = 0.75 m, respectively). Multiple vortices in a cross section could make the microalgal cells move from one half of the pipe to the other half more frequently, which facilitates the enhancement of the L/D cycles.

The vortex intensity in IN15 is lower than that in H20 (Fig. 6c), but the L/D cycle frequency of IN15 is higher than that of H20. That is, a weaker vortex flow generates more L/D cycles. This result indicates that when the radial height is decreased by 5 mm, the relocation of vortices closer to the L/D boundary has a more important impact on the increase in the L/D cycle frequency, and this impact may offset the adverse effect of the weak vortex intensity. However, when the radial height is decreased by 10 mm (the IN10 case), the result is much different. Figure 7a shows that *f*_av_ of IN10 is lower than that of H20 under a wide range of incident light intensities (from 375 μmol m^−2^ s^−1^ to 2400 μmol m^−2^ s^−1^). This result is because the vortex is further weakened as the mixer height is further reduced (Fig. 7c), and the increase in *f*_av_ through the relocation of the vortices and the multivortex structure in IN10 is less than the decrease in *f*_av_ caused by the decrease in the vortex intensity. Thus, for the PBR in this study, a decrease in the mixer’s radial height from the inner side can actually increase the L/D cycle frequency, but too much of a decrease in the radial height from the inner side may worsen the L/D cycle frequency. There is a balance between the positive and negative impacts.

In addition, Fig. 7a shows that as the incident light intensity increases, the difference between the L/D cycle frequency of IN15 and that of H20 first increases and then decreases. That is, the advantage of decreasing the mixer’s radial height from the inner side in enhancing the L/D cycle frequency is obvious at moderate incident light intensities and is not obvious at very low or very high incident light intensities among the range of 375–2400 μmol m^−2^ s^−1^. This finding is reasonable because at very low incident light intensities (e.g., 375 μmol m^−2^ s^−1^), the L/D boundary is very close to the wall of the pipe, and the vortex cores in H20 and IN15 are both far away from the L/D boundary despite the relocation of the vortex core in IN15. As a result, the *f*_av_ values for both H20 and IN15 are very low. As the incident light intensity increases (e.g., 800 and 1200 μmol m^−2^ s^−1^), better synergy can be found in IN15 than that in H20, and consequently, *f*_av_ of IN15 is much higher than that of H20. As the incident light intensity increases further to a very high value (e.g., 1600, 2000 and 2400 μmol m^−2^ s^−1^), the L/D boundary approaches the core region of the pipe. Under these conditions, the effect of relocating a vortex far from the core region on enhancing L/D cycles in the case of IN15 is weakened. In large-scale outdoor microalgae cultivation, the incident light intensity depends on the natural sunlight conditions (e.g., 1200 μmol m^−2^ s^−1^ in the Neimenggu municipality in China [10], and this value is in the domain of moderate incident light intensity shown in Fig. 4c). In this situation, decreasing the mixer’s radial height from the inner side is an appropriate way to increase the L/D cycle frequency.

In contrast, the results for H20, EX15 and EX10 shown in Fig. 7a demonstrate that as the mixer’s radial height decreases from the outer side, *f*_av_ decreases under a wide range of incident light intensities (from 375 to 2400 μmol m^−2^ s^−1^). The reason is as follows: On the one hand, after the vortices in EX10 and EX15 have merged into a single vortex near *z* = 0.79 m and 0.75 m, respectively, this vortex becomes stable, and its core is located in the center region of the pipe (Fig. 6a, b), which is far from the L/D boundaries for incident light intensities from 375 to 2400 μmol m^−2^ s^−1^ (Fig. 4). Thus, the vortex has a weak effect on the enhancement of the movement of the microalgal cells across the light and dark zones. On the other hand, the intensity of the vortices decreases as the radial height of the mixer decreases from the outer side (Fig. 4c). This finding indicates that reducing the radial height of the helical mixer from the outer side is not an effective way to increase the L/D cycle frequency.

##### Pressure drop

As shown in Fig. 7b, the pressure drop in the PBR decreases as the radial height of the mixer decreases as a whole. This finding is reasonable since the vortex flow and turbulence are weakened as the radial height of the mixer decreases, and thus, the friction factor decreases. According to Darcy’s law, the pressure loss consequently decreases. The pressure drop in EX15 (1209.5 Pa) is lower than that in IN10 (1284.4 Pa), while the radial height of the latter is higher than that of the former. This result means that decreasing the radial height from the outer side may save more friction loss compared with decreasing the radial height from the inner side.

Combining the results in “L/D cycle frequency” section, it can be found that decreasing the radial height from *H *= 20 mm to *H *= 15 mm from the inner side can not only increase the L/D cycle frequency but also decrease the pressure drop in the PBR, and this finding means that more L/D cycles are generated while less pumping costs are consumed. In view of the synergy idea, decreasing *H* from 20 to 15 mm not only relocates the vortex closer to the L/D boundary but also weakens the vortex intensity and thus lowers the pumping costs. This result is meaningful since it provides a possible way to enhance L/D cycles and reduce pumping costs simultaneously.

##### Efficiency of L/D cycle enhancement

To evaluate changes in L/D cycle frequency and pressure drop caused by a mixer, the efficiency of L/D cycle enhancement, *η*, is shown in Fig. 7c. The efficiency of IN10 or IN15 is higher than that of H20 for a wide range of incident light intensities (375–2400 μmol m^−2^ s^−1^), verifying that reducing the radial height from the inner side works well. Additionally, the efficiency of IN15 is higher than that of EX10 and EX15, indicating that reducing the radial height from the inner side works better than reducing the radial height from the outer side.

## Conclusions

The synergy idea indicates that improving the synergy between flow and light fields can markedly enhance the L/D cycle frequency with a lower increase in pumping costs, which is favorable for practical applications. We can obtain better synergy if the vortex core and L/D boundary are closer to each other and the vortex whose core is too far from the L/D boundary is removed. With such an idea, we can not only have a deeper understanding of some known numerical and experimental results about mixer addition but also develop useful rules to guide the design of mixers. The geometrical parameter design of a helical mixer is taken as an example to illustrate the importance and feasibility of the synergy idea. By applying the method of relocating vortices closer to the L/D boundary, which is accomplished by reducing the mixer’s radial height from the inner side, the L/D cycle frequency of the PBR is increased by up to 10.8% for incident light intensities ranging from 375 to 2400 μmol m^−2^ s^−1^, and the pumping costs are simultaneously decreased by 23.8%.

## Abbreviations

L/D: light/dark; PBR: photobioreactor.

### List of symbols

*D*: inner diameter of the pipe [m]; *E*: pumping cost per unit time [J s^−1^]; *E*_0_: pumping cost per unit time of a plain PBR [J s^−1^]; *f*: L/D cycle frequency [Hz]; *f*_av_: average L/D cycle frequency [Hz]; *f*_av,0_: average L/D cycle frequency of a plain PBR [Hz]; *H*: radial height of the helical mixer [m]; ID: serial number of a particle [–]; *I*_0_: incident light intensity [μmol m^−2^ s^−1^]; *J*_ABS_: absolute vorticity flux [s^−1^]; *n*: number of L/D cycles of a particle [–]; *N*: number of particles [–]; *P*: total pressure [Pa]; *P*_up/down_: average total pressure at the upstream/downstream surface of the mixer [Pa]; *P*_s,local_: local static pressure at the inlet/outlet surface [Pa]; *P*_d,local_: local dynamic pressure at the inlet/outlet surface [Pa]; *S*: cross-sectional area of the pipe [m^2^]; *t*_c_: duration of the L/D cycle [s]; *t*_d_: dark duration [s]; *t*_l_: light duration [s]; *U*_av_: average inlet velocity [m s^−1^]; *x, y, z*: Cartesian coordinates [m]; *y*^+^: nondimensional distance from the cell centers of the first layer grid to the wall [–].

### Greek symbols

*η*: efficiency of L/D cycle enhancement [–]; *ϕ*: volumetric flow rate [m^3^ s^−1^]; *ω*_z_: axial component of vorticity [s^−1^].

## Additional file

**Additional file 1.** Independent validation of the maximum tracking time and verification of the number of tracked particles.
