# Supplementary material for: Synergy between flow and light fields and its applications to the design of mixers in microalgal photobioreactors
Source: Biotechnol Biofuels. 2019 Apr 23;12:93. doi: 10.1186/s13068-019-1430-y (PMC6477735; doi:10.1186/s13068-019-1430-y)
Supplement: Supplementary file 1 — Additional file 1. Independent validation of the maximum tracking time and verification of the number of tracked particles. [file 13068_2019_1430_MOESM1_ESM.docx]

# Additional file 1. Independent validation of the maximum tracking time and verification of the number of tracked particles

**Fig. S1.** Independent validation of the maximum tracking time and verification of the number of tracked particles: (a) the number of particles that escape from the PBR outlet under different maximum tracking times when 1000 particles are released simultaneously at *z* = 0.3 m and (b) the impact of the number of statistical particles on *f*_av_ (*I*_0_=375 𝜇mol m^-2^ s^-1^) when the maximum tracking time is 10 s.

As recommended for the reliability of statistical data (Ref. [3] in the manuscript) as well as taking the computational cost into account, 1000 particles are released simultaneously from the center of the pipe with 0 m/s velocity at *z* = 0.3 m. In this situation, when the maximum tracking time is more than 10 s, the number of particles passing through the surface of *z* = 1.5 m remains nearly constant for each case (H0, IN10, IN15, EX10, EX15 and H20), as shown in Fig. S1 (a). Therefore, the maximum tracking time is taken as 10 s in this work. On the other hand, when the maximum tracking time is 10 s, *f*_av_ becomes stable when the number of statistical particles is larger than 900, as shown in Fig. S1 (b), and this finding verifies that taking the number of tracked particles as 1000 is reasonable.
